# Successful Reduction of a Luxation of the Shoulder Joint of Fifty One Days Standing

**Published:** 1838-01-31

**Authors:** Thomas Harris

**Affiliations:** Surgeon to the Hospital


### CLINICAL REPORTS.

### PENNSYLVANIA HOSPITAL.

*Successful reduction of a luxation of the shoulder joint of fifty one days standing; by* Thomas Harris, *M. D. Surgeon to the Hospital.*

R. H. a native of Virginia, aged thirty-seven years, a robust, healthy man, a wheelwright by trade, was admitted into the Hospital, 17th January 1838. While riding on the night of the 30th November last, his horse stumbled, and he was thrown with violence against the root of a tree; this produced a luxation of the head of the right humerus downwards into the axilla, with much contusion. He neglected to apply for surgical assistance, for a month, as he "supposed that his shoulder was merely bruised." On the 2d of January, he showed his arm to a physician in the neighbourhood, who at once pronounced it to be a luxation. He states, that three unsuccessful attempts at reduction were made, according to the usual approved methods, without the use of pulleys however: these attempts had been preceded each time by copious venesection, as was indicated by his blanched countenance. At the time of his admission, he complained of great numbness down his arm; the shoulder presented an unusual appearance of squareness, owing to the wasting away of the deltoid muscle; the head of the bone was to be felt very distinctly in the axilla, and the artery could be traced lying directly upon it.

*January 20th*, he was brought into the amphitheatre, present Drs. Harris, Randolph, and Norris. Dr. Harris prefaced the operation by remarking, that he was not altogether certain of success, that he would make no violent efforts, as a rupture of the artery or severe laceration of the soft parts might take place. He intended using a moderate degree of force with pulleys, and keeping it up for a long time, opening at the same time a vein, and nauseating the man with tartar emetic. No benefit, he thought, was to be anticipated from the warm bath, as the relaxation of the muscles which it occasions, would pass off, before extension could be applied. He would attempt no circumduction of the limb, as has been sometimes recommended, nor make any violent effort to break up adhesions: he would rely entirely on gradual extension, kept up, without intermission, for a considerable time.

Fifteen minutes previous to the operation, half a grain of tartar emetic had been administered. The man was now placed upon a low stool; a folded sheet was passed under the axilla of the injured side, and secured to a post on his left hand. A roller towel was firmly secured on the upper and under side of the arm from the elbow to within two and a half inches of the axilla, by a wet roller, and the pulleys were applied so as to draw the arm outwards and slightly upwards. The acromion process of the scapula was fixed by a towel passing over the edge of it and down to the floor on the opposite side, where it was held by an assistant. A vein was now opened, two grains of tartar emetic were given, and a moderate degree of force applied to the pulleys. In eight minutes, without taking off the pulleys, an attempt was made to replace the bone, by fixing the knee in the axilla, and pressing the elbow downward, but without success. A towel was now passed under the axilla, and tied over the top of the shoulder; an assistant, standing over him on a chair, drew the arm strongly upward by this. Two grains of tartar emetic were now given, and, at the end of ten minutes, the knee being placed in the axilla, the pulleys were cast off, and the elbow drawn down to the side. The bone went into its place, but without any noise: this was owing to the previous overstretching of the muscles. From the prostrated condition of the patient, which prevented any action in the muscles, and the wasting of the deltoid, there were some doubts raised, whether the reduction was effected. Dr. Harris stated, that he had frequently seen the same appearance of flatness in the shoulder, after the bone was replaced, when it had been for a long time dislocated. By holding the acromion with one hand, and pressing the elbow forcibly upwards, the head of the bone could be felt in its natural situation, though when left to itself, the weight of the arm drew it downwards fully an inch. The clavicle bandage of the house* was now applied, and the patient put to bed. He had lost in all ʃ℥xx. of blood. In the afternoon, he complained of agonizing pain in the elbow; ʒss. of laudanum was administered, and repeated every two hours, until ʒii. were taken, when he fell asleep. With the last dose of laudanum, he was allowed ℥ii. of brandy.

*Sunday*, 21*st*.—Complained of but little pain. Soap liniment and laudanum to the shoulder. A dose of rhubarb and magnesia. Absolute rest. The shoulder had now regained a nearly natural appearance, the flatness having disappeared.

*Saturday*, 27*th*.—The man is doing very well, he intends returning home in a few days.

[The operation lasted twenty minutes.]

*For description of this, see No. 2, page 23, (Wallace on Fractures.)

